# Current State of Precision Medicine in Primary Systemic Vasculitides

**DOI:** 10.3389/fimmu.2019.02813

**Published:** 2019-12-17

**Authors:** Erkan Demirkaya, Zehra Serap Arici, Micol Romano, Roberta Audrey Berard, Ivona Aksentijevich

**Affiliations:** ^1^Division of Paediatric Rheumatology, Department of Paediatrics, Schulich School of Medicine & Dentistry, University of Western Ontario, London, ON, Canada; ^2^Department of Paediatric Rheumatology, Sanliurfa Training and Research Hospital, Sanliurfa, Turkey; ^3^Department of Pediatric Rheumatology, Istituto Ortopedico Gaetano Pini, Milan, Italy; ^4^Inflammatory Disease Section, National Human Genome Research Institute, National Institutes of Health, Bethesda, MD, United States

**Keywords:** precision medicine, vasculitis, vasculitides, genome-wide association studies, epigenetics, extracellular vesicles, monogenic systemic autoinflammatory diseases

## Abstract

Precision medicine (PM) is an emerging data-driven health care approach that integrates phenotypic, genomic, epigenetic, and environmental factors unique to an individual. The goal of PM is to facilitate diagnosis, predict effective therapy, and avoid adverse reactions specific for each patient. The forefront of PM is in oncology; nonetheless, it is developing in other fields of medicine, including rheumatology. Recent studies on elucidating the genetic architecture of polygenic and monogenic rheumatological diseases have made PM possible by enabling physicians to customize medical treatment through the incorporation of clinical features and genetic data. For complex inflammatory disorders, the prevailing paradigm is that disease susceptibility is due to additive effects of common reduced-penetrance gene variants and environmental factors. Efforts have been made to calculate cumulative genetic risk score (GRS) and to relate specific susceptibility alleles for use of target therapies. The discovery of rare patients with single-gene high-penetrance mutations informed our understanding of pathways driving systemic inflammation. Here, we review the advances in practicing PM in patients with primary systemic vasculitides (PSVs). We summarize recent genetic studies and discuss current knowledge on the contribution of epigenetic factors and extracellular vesicles (EVs) in disease progression and treatment response. Implementation of PM in PSVs is a developing field that will require analysis of a large cohort of patients to validate data from genomics, transcriptomics, metabolomics, proteomics, and epigenomics studies for accurate disease profiling. This multi-omics approach to study disease pathogeneses should ultimately provide a powerful tool for stratification of patients to receive tailored optimal therapies and for monitoring their disease activity.

## Introduction

Precision medicine (PM) can be defined as tailored medical care that is primarily based upon understanding the molecular sequence of biologic events causal to disease and critical for diagnosis and therapy. The term precision medicine should be distinguished from the term personalized medicine whereby prevention and treatment are being developed uniquely for each patient, although these two terms are used interchangeably (1). Both communicate a concept of individualized medicine, although in PM, the focus is on identifying health care approaches that will be effective for a group of patients who share similar genetic, environmental, and lifestyle disease susceptibility factors. Recognizing subgroups of patients with similar disease risk factors will ensure that they will receive optimal treatment to improve their quality of life and health outcomes.

Primary systemic vasculitides (PSVs) (Table 1) are a heterogeneous group of diseases in their etiology, clinical presentation, and response to therapy. Severity and location of symptoms vary greatly, and most classification schemes that attempt to advance clinicians the ability to diagnose and treat patients, are based on blood vessel size, autoantibodies profile [e.g., anti-neutrophil cytoplasmic antibody (ANCA)], and histopathological findings. In addition to genetic predisposition, there is a role for environmental factors, including exposure to drugs and infectious agents, in the pathogenesis, and prognosis of PSVs. The past decade has witnessed major advances in genetics research that have improved our understanding on molecular mechanisms in PSVs. New sequencing technologies and high-throughput genotyping arrays used for genome-wide association studies (GWAS) generated vast amount of data that will require validation by meta-analysis in large cohorts of patients. Discovery of monogenic diseases mimicking PSVs offered additional insights on biochemical pathways that are shared between rare and more common forms of vasculitis. Recent advances in epigenetic research support the role of extracellular vesicles (EVs) in the pathogenesis of PSVs, which could be used for developing novel biomarkers to monitor disease activity, prognosis, and treatment outcomes.

**Table 1:** Chapel Hill Consensus Conference Nomenclature (CHCC 2012) of vasculitides (2).

Large-vessel vasculitis (LVV)
Takayasu arteritis (TAK) Giant cell arteritis (GCA)
Medium-vessel vasculitis (MVV)
Polyarteritis nodosa (PAN) Kawasaki disease (KD)
Small-vessel vasculitis (SVV)
Antineutrophil cytoplasmic antibody (ANCA)–associated vasculitis (AAV) ° Microscopic polyangiitis (MPA) ° Granulomatosis with polyangiitis (Wegener's) (GPA) ° Eosinophilic granulomatosis with polyangiitis (Churg-Strauss) (EGPA) Immune complex SVV ° Anti–glomerular basement membrane (anti-GBM) disease ° Cryoglobulinemic vasculitis (CV) ° IgA vasculitis (Henoch-Schönlein purpura) (IgAV/HSP) ° Hypocomplementemic urticarial vasculitis (HUV) (anti-C1q vasculitis)
Variable vessel vasculitis (VVV)
Behçet's disease (BD) Cogan's syndrome (CS)
Single-organ vasculitis (SOV)
Cutaneous leukocytoclastic angiitis Cutaneous arteritis Primary central nervous system vasculitis Isolated aortitis Others
Vasculitis associated with systemic disease
Lupus vasculitis Rheumatoid vasculitis Sarcoid vasculitis Others
Vasculitis associated with probable etiology
Hepatitis C virus–associated cryoglobulinemic vasculitis Hepatitis B virus–associated vasculitis Syphilis-associated aortitis Drug-associated immune complex vasculitis Drug-associated ANCA-associated vasculitis Cancer-associated vasculitis Others (e.g., varicella zoster virus–associated vasculitis)

Herein, we discuss the up-to-date knowledge on the genetic predisposition to PSVs and review recent clinical research studies aimed to further our understanding of the pathogeneses and outcomes of vasculitides.

## GWAS in PSVs

GWAS have been used, following the completion of the Human Genome project in 2003 and the International HapMap Project in 2005, to identify association of common and reduced-penetrance variants, termed single nucleotide polymorphisms (SNPs), with human traits or diseases (3). The primary goal of GWAS has been to elucidate the biology of polygenic and complex human diseases, which can be translated toward the development of novel therapeutics. GWAS have also been used to study gene-environment interactions, response to therapies, and more recently for risk prediction. GWAS are based on the genotyping of large cohorts of patients and ancestry-matched controls for SNPs in the entire genome. These associations are tested with gene variants that have a minor allele frequency (MAF) higher than 5% in the general population and are considered significant at the *p*-value genome-wide threshold of 5 × 10^−8^ (4). For a number of autoimmune disorders, SNPs in the human leukocyte antigen (HLA) [also known as major histocompatibility complex (MHC)] and genes encoded within the locus have been shown to play a major role in susceptibility to disease (4). In primary vasculitides (PSVs), GWAS have been performed primarily in Kawasaki disease (KD) and Behçet's disease (BD) (Table 2) (5–42, 44–46).

**Table 2:** Genome-wide association studies (GWAS) in primary systemic vasculitides (PSVs).

**Related risk and references**	**Genes from GWAS**	**Therapeutic implications**
**Kawasaki Disease (KD)**		
Susceptibility genes for KD (5–16)	*HLA, HCP5, FCGR2A, BLK*, SLC8A1, *CD40, NMNAT2, DAB1, COPB2, NAALADL2, IGHV, ZFHX3, NFKBIL1, ERAP1*, EBF2, CACNB2, *LTA*, and *LEF1*	SNP in SLC8A1 (calcium signaling pathway) can be proof for using calcineurin inhibitors in KD
Susceptibility genes for cardiovascular disease in KD (8, 12, 17–22)	*TIAM1, NEBL, PLCB4*/*PLCB1, TUBA3C*, SLC8A1, *PELI1, KCNN2, TIFAB*, and *AGT*	
Susceptibility genes for intravenous immunoglobulin (IVIG) resistance in KD (23, 24)	*BCL2L11* and *SAMD9L*	BCL2L11 and SAMD9L: prediction of IVIG resistance
**Behçet's Syndrome (BS)**		
Susceptibility genes for BS (25–34)	*HLA-B51, STAT4, IL10, GIMAP, IL23R-IL12RB2, CCR1, ERAP1, KLRC4, FUT2, IL12A, NAALADL2, YIPF7, CPVL, UBAC2, LOC100129342, UBASH3B*, and *KIAA1529*	Inhibition of IL-12/IL-23 pathway activation may be a treatment target
**Antineutrophil cytoplasmic antibody (ANCA)-associated vasculitis (AAV)**		
Related with anti-MPO AAV (35)	*HLA-DQ*	
Related with anti-PR3 AAV (35, 36)	*HLA-DP, SERPINA1*, and *PRTN3*	
Related with AAV (36, 37)	PTPN22, *SEMA6A*	
**Takayasu Arteritis (TAK)**		
Susceptibility genes for TA (38–41)	*HLA-B/MICA, HLA-DQB1/HLA-DRB1, FCGR2A/FCGR3A, IL12B, RPS9/LILRB3, IL6, PTK2B, LILRA3/LILRB2, DUSP22, KLHL33, HSPA6/FCGR3A*, the intergenic locus on chromosome *chromosome21q.22, MICB*, and HLA-B*52 (poor prognosis)	
**Immunoglobulin A Vasculitis/Henoch-Schönlein Purpura (IgAV/HSP)**		
Susceptibility locus for IgAV/HSP (42)	HLA-DRB1	
**Giant cell arteritis (GCA)**		
Susceptibilty genes for GCA (43)	HLA-DRB1*04, PLG, and P4HA2	

### Kawasaki Disease

KD is an acute, self-limited vasculitis that typically affects infants and children under the age of 5 years. Coronary artery aneurysms (CAAs) occur in 25% of untreated patients and may lead to ischemic heart disease, myocardial infarction, and sudden death at a young age. The pathogenesis of KD remains unknown; however, it is thought that host genetics play an important role in susceptibility and disease outcome. Interestingly, the incidence of KD is up to 50-fold higher in children of Asian descent. Epidemiologic and clinical features of KD also strongly support an infectious etiology in genetically predisposed children (47).

GWAS in KD have identified a number of susceptibility SNPs/genes that contribute to the risk of KD (*HLA, FCGR2A*, SLC8A1, ZFHX3, NMNAT2, *BLK, DAB1, HCP5, COPB2, ERAP1, NAALADL2, CD40, NFKBIL1, IGHV, EBF2, CACNB2, LTA*, and *LEF1*) (5–16) to the risk of cardiovascular disease in KD (*TIAM1, NEBL, PLCB4*/*PLCB1, TUBA3C, SLC8A1, PELI1, KCNN2, TIFAB*, and *AGT*) (8, 12, 17–22) and to the risk of intravenous immunoglobulin (IVIG) resistance (*BCL2L11* and *SAMD9L*) (23, 24). Involvement of the HLA region in susceptibility to KD has been controversial and has not been replicated across different ancestral groups.

Shimizu et al. (12) identified three functional SNPs in the solute carrier family 8, member 1 (*SLC8A1)* gene to be associated with susceptibility to KD in Japanese and European cohorts (meta analysis *p* = 0.0001). *SLC8A1* encodes NCX1 (a sodium/calcium exchanger) that functions as a bidirectional sodium/calcium channel. Patients homozygous for the risk allele (rs13017968) have higher rates of coronary artery abnormalities. Homozygosity for rs13017968 is associated with an increase in Ca^2+^ flux in EBV-transformed B cells of healthy individuals. The NCX1 protein expression was detected in the postmortem coronary artery tissue of a young KD patient. Another study by Onouchi et al. (48) found a coding SNP (rs3741596) in the ORAI Calcium Release-Activated Calcium Modulator 1 (*ORAI1*) gene to be significantly associated with KD in Japanese patients (meta analysis *p* = 0.00041). Interestingly, frequency of the risk allele is more than 20 times higher in Japanese compared to Europeans, which may account for higher prevalence of KD in the Japanese population. Together, these genetic and functional data provide evidence for the role of Ca^2+^-mediated signaling pathways in the pathogenesis of KD and for the use of calcineurin inhibitors (49).

Lv et al. (46) used statistically significant candidate variants from multiple GWAS and other gene association studies for pathways analysis. This investigation showed that KD susceptibility genes are enriched in functional networks for calcium ion homeostasis and immune responses and highlighted the role of nuclear transcription factor of activated T cells (NF-AT) and nuclear factor (NF) kappa light chain enhancer of activated B cells (NF-κB) in the pathogenesis of KD.

Another indication from GWAS for the use of new therapies in KD has come from the study by Chang et al. (44). The promoter variant, rs2736340, in the B lymphoid tyrosine kinase (*BLK*) gene was significantly associated with susceptibility to KD in multiple Asian populations [odds ratio (OR) = 1.498, 1.354–1.657; *p* = 4.74 × 10(−31)]. The transformed and primary B cells with the risk allele express significantly lower levels of BLK and have reduced signaling downstream of B cell receptors. These data suggest a role for humoral immunity in the pathogenesis of the acute stage of KD (44). Although B cells and autoantibodies have been found in blood samples of KD patients, their role, whether they are causal or bystanders of an activated immune system or specific to an infectious agent in the etiology of KD, is currently unknown (50).

Standard treatment for KD consists of a single infusion of high-dose IVIGs and high-dose aspirin (until the fever has resolved); however, the mechanism of action remains elusive. Because IVIG therapy is the mainstay of treatment for KD, there has been interest to study the effect of SNPs and copy number variants (CNVs) on the function of receptors for IgG, the Fc-gamma receptors (FcγRs). There are five genes encoding the low-affinity FcγRs (*FCGR2A, FCGR2B, FCGR2C, FCGR3A*, and *FCGR3B)* that are clustered in the *FCGR2/3* locus. Recently, Nagelkerke et al. published a study that evaluated the previously reported genetic association with the rs1801274 variant in this gene locus. The *FCGR2A* coding SNP rs1801274 has been validated in a number of KD ancestry cohorts and was shown to cause a difference in the ability of FcγRIIa to bind the human IgG2 immunoglobulin isotype. This new study defined haplotype blocks (set of closely linked alleles/markers that are inherited together) across the *FCGR2/3* gene locus in over 4,000 individuals from different populations. The FCGR2C-ORF haplotype has stronger susceptibility to KD in the European cohort than the previously reported risk allele rs1801274. However, the FCGR2C-ORF haplotype is extremely rare in Asians and thus cannot explain the high prevalence of KD in Asian populations. This research points out the importance of proper interpretation of genetic association studies in the context of patients' ancestry (51).

Kuo et al. developed a genetic model to predict IVIG resistance in KD. They identified 11 candidate SNPs in *ADTRP, KLF6, EX0C2, ZNF438-ZEB1*, and *MIR548A3-ZPLD1* genes to calculate a weighted genetic risk score (wGRS) in 126 IVIG responders and 24 IVIG non-responders (52). Patients with IVIG resistance had higher wGRS scores than individuals who were IVIG responsive; however, the wide confident intervals (CIs) for the OR suggest that these findings should be replicated in larger cohorts of patients.

Kim et al. (24) recently identified a new gene locus, *SAMD9L*, that is associated with IVIG resistance in Korean and Japanese KD cohorts. The meta-analysis of IVIG non-responders (*n* = 317) and IVIG responders (*n* = 1221) showed the association of the common intronic variant (rs28662; MAF = 13% in gnomAD) with IVIG resistance [(OR) = 4.30, *p* = 5.3 × 10^−6^]. This SNP was predicted to change the motif for transcription factor binding site and was also associated with differential expression of the *SAMD9L* gene in lymphoblastoid cell lines. Previously, variants in *SAMD9L* have been associated with susceptibility for systemic lupus erythematosus, systemic sclerosis, and other immune-related diseases, such as juvenile myelomonocytic leukemia, acute myeloid leukemia, myelodysplastic syndrome, hepatitis-B-related hepatocellular carcinoma, and ataxia-pancytopenia syndrome. However, the role of SAMD9L in immune signaling pathway is unclear (24).

### Behçet's Disease

BD is a chronic multisystem inflammatory disorder characterized by recurrent oral/genital ulcers, ocular involvement, skin lesions/pathergy, presenting with remissions and exacerbations. The disease is common in populations living along the ancient Silk Road: Turkish, Iranian, Chinese, Korean, and Japanese. Classic BD is a complex disease with a strong genetic predisposition. Although the etiology of BD remains unclear, recent immunogenetic findings from GWAS provide clues to its pathogenesis (53, 54).

HLA-B*51 remains the most significant genetic susceptibility factor for BD in multiple populations; however, it accounts for <20% of the genetic risk (55, 56). The ongoing controversy is about whether the disease association with HLA-B*51 is attributed to a role of this variant itself or is due to its linkage disequilibrium (LD) with another variant in the region. There are also other BD disease-associated amino acids variants in the HLA-B locus that are thought to reside within the antigen-binding grove of the molecule (57). The association between specific MHC class I alleles and certain disease manifestations have been reported (58–61). A meta-analysis based on 72 studies in 74 study populations revealed moderate association of HLA-B*51/B5 with male gender and high prevalence of eye and mucocutaneous involvement (60).

GWAS have identified a number of population-specific associations in the following genes: *IL23R-IL12RB2, IL10, TNFAIP3, STAT4, CCR1-CCR3, KLRC4, ERAP1*, and *FUT2*. Variants in some genes, including *KIAA1529, UBASH3B, GIMAP, IL12A, NAALADL2, YIPF7, CPVL, LOC100129342*, and *UBAC2*, did not reach genome-wide significance (*p* < 5.0 × 10^−8^) (25–34). Most of these alleles act as susceptibility factors, while some variants appear to be protective. BD-risk alleles in the *IL10* susceptibility gene locus were the first variants identified outside of the HLA region by GWAS in Turkish and Japanese populations (29, 30). The SNP rs1518111 was subsequently replicated in Middle Eastern Arab, Greek, British, Korean, Iranian, and Han-Chinese samples and is today the only variant consistently associated with susceptibility to BD across all ancestries (57).

More recent studies investigated whether there is evidence for interaction (epistasis) among BD susceptibility genes. The endoplasmic reticulum (ER)-associated protease (ERAP1) haplotype tagged by the coding variant, rs17482078 (p.Arg725Gln), has a large effect with an OR of 3.78 in patient carriers for HLA-B*51. Interestingly, the same variant rs17482078 is protective for psoriasis and ankylosing spondylitis (AS). ERAP1 is an enzyme that clips proteasome-processed peptides in the ER before these peptides are loaded on class I HLA molecules (56). The epistasis observed between HLA-B*51 and the risk haplotype of *ERAP1* suggests that an HLA class I-peptide presentation contributes to BD. The difference between risk and protection conferred by the *ERAP1* haplotype may be explained by altered binding affinities among specific peptides, trimmed or not trimmed by the ERAP1 variant, for different HLA class I molecules (62).

Dense genotyping of immune-related loci extended the list of susceptibility genes to include risk alleles in *IL1A-IL1B, IRF8, CEBPB-PTPN1*, and *ADO-EGR2* genes in the Turkish set of 1,900 cases and 1,779 controls. This study implicated the innate immune response to microbial exposure in susceptibility to BD (63). Bakir-Gungor et al. reported new BD-associated common variants in pathways, including focal adhesion, complement and coagulation cascades, mitogen-activated protein kinases (MAPK) signaling, proteasome pathways, extracellular matrix (ECM)–receptor interaction, and transforming growth factor-β (TGFβ) signaling both in Japanese and Turkish populations (64).

Targeted sequencing of candidate immunological genes has revealed the contribution of rare non-synonymous variants of *NOD2, TLR4, IL23*, and *MEFV* in the BD pathogenesis. In particular, heterozygosity for a common high-penetrance familial Mediterranean fever (FMF)-associated mutation p.Met694Val is significantly associated with BD risk in the Turkish population, although not in Japanese patients due to its absence in the Asian populations (65). *MEFV* encodes pyrin, which regulates caspase-1-mediated interleukin (IL)-1β production.

Nakano et al. investigated the functional effect of GWAS-identified BD risk alleles in *IL-10* and *CCR1*. They observed a differential protein expression of IL10 and CCR1 in M1 vs. M2 macrophages (Mϕ) derived from BD patients. BD skin lesions showed predominance of M1 Mϕ. This study suggests that targeting pathways and genes that play a role in the Mϕ polarization should be explored in the treatment of BD (66).

Taken together, GWAS have implicated a number of genes of the innate and adaptive immunity with increased susceptibility to BD. Whether inhibition of these molecular pathways will be effective in the treatment of BD remains to be seen.

### ANCA-Associated Vasculitis (AAV)

AAV is a heterogeneous group of disorders characterized by necrotizing inflammation of small vessels and by the presence of ANCA. The two most common ANCAs are the autoantibodies that target the proteins: myeloperoxidase (MPO) and proteinase 3 (PR3). AAV includes the clinical syndromes of microscopic polyangiitis (MPA), granulomatosis with polyangiitis (GPA), and eosinophilic granulomatosis with polyangiitis (EGPA). ANCA directed against PR3 are preferentially associated with GPA, and anti-MPO is associated mainly with MPA and EGPA. Although the role of ANCA in the pathogenesis of AAV is appreciated, the mechanism for ANCA-mediated development of AAV is not clear. Genetic predisposition and exposure to environmental factors, such as drugs and infectious agents, are possible triggers of excessive activation of neutrophils and injury to small blood vessels. The clinical signs vary and may affect several organs, such as the kidney, intestine, lung, and skin.

Currently, three GWAS have been performed in AAV. These studies identified risk alleles in *HLA-DQ, HLA-DP, HLA-DR, SERPINA1, PTPN22, PRTN3*, and *SEMA6A* genes as contributing to AAV (35–37). A meta-analysis of 140 reported genetic variants associated with AAV distinguished the most significant 33 alleles in 15 genes (67). Twenty of these 33 genetic variants were present in the HLA region, suggesting an important role for the antigen presentation in the disease pathogenesis. Two GWAS showed that the SNP association signal in the HLA region was fully accounted for by HLA-DPB1*04 allele (35, 37).

Lyons et al. established that MPA and GPA are two distinct diseases based on their genetic profile (35). Anti-MPO positive AAV (MPA) is strongly associated with the HLA-DQ, whereas anti-PR3 AAV (GPA) is associated with the HLA-DP region and the genes encoding α(1)-antitrypsin (*SERPINA1*) and serine proteinase 3 (*PRTN3*) (35, 68). SERPINA1 is a major inhibitor of PR3, and lower levels of α (1)-antitrypsin can lead to higher levels of circulating PR3 and higher production of anti-PR3 autoantibodies. Population difference in HLA-class II genotype frequency plays a role in the epidemiology of this disease. Among AAVs, GPA and PR3-AAV are prevalent in European populations, whereas MPA and MPO-AAV are predominant in the Japanese population. In the North American population, the HLA- DPB1*04 has the strongest association with GPA, whereas in the Japanese population, HLA- DRB1*09:01 is strongly associated with MPA (68–70).

Other notable variants outside the HLA region include alleles in protein tyrosine phosphatase non-receptor type 2 (*PTPN22*) gene, which negatively regulates the production of an immunosuppressive cytokine interleukin (IL-10) (71). The gain-of-function risk allele, p.Arg260Trp, is linked to GPA-AAV in addition to a number of other autoimmune diseases (72, 73). A non-coding SNP in the vicinity of *SEMA6A* is linked to GPA in the GWAS study of Caucasian cohort of 528 patients and at a genome-wide signficance (*p* = 2.09 × 10^−8^). The *SEMA6A* gene codes for semaphorin 6A and is highly expressed in dendritic cells; however, its function is unclear (37).

Lower and higher copy number variants in the *FCGR3B* gene and a higher *DEFB4* gene copy number have been associated with risk for AAV (74–76). A possible relationship between the *FCGR3B* copy number and susceptibility to AAV needs to be clarified experimentally (77).

Lee et al. performed gene-ontology (GO) enrichment and protein–protein interaction (PPI) networks analysis of significant risk alleles to identify the key pathophysiological pathways in the pathogenesis of AAV. The most significant pathways include the processing of antigens *via* HLA class II, T-cell receptor signaling, and interferon (IFN)-γ mediated signaling pathways (78).

### Takayasu Arteritis (TAK)

TAK is a large-vessel vasculitis that predominantly affects young women and can cause organ-threatening ischemia, such as pulseless limbs, aortic regurgitation, pulmonary infarct, and kidney failure by occluding the aorta and/or its branches. TAK is seen all over the world, but Eastern Asia, especially Japan, has the highest incidence. The etiology of TAK is elusive, but genetic predisposition is likely. Corticosteroids, anti-tumor necrosis factor (TNF)-α, anti-IL-6, and abatacept are used for induction of remission and with aim to prevent relapse.

GWAS have shown that *HLA-B/MICA, HLA-DQB1/HLA-DRB1, FCGR2A/FCGR3A, IL12B, RPS9/LILRB3, IL6, PTK2B, LILRA3/LILRB2, DUSP22, KLHL33*, and *HSPA6/FCGR3A* and the intergenic locus on chromosomes *21q.22, MICB*, and HLA-B*52 are susceptibility gene loci in TAK (38–41). Risk alleles in *HLA-B/MICA* and *HLA-DQB1/HLA-DRB1* were identified in Turkish and European cohorts of patients. Other notable associations are in *PSMG1, IL12*, and *IL23* gene loci but not at the level of genome-wide significance (39). HLA-B*52 allele is related to poor prognosis in TAK (79, 80). Risk alleles in the *RPS9/LILRB3* locus and *IL6* may have immunoregulatory effects (38). The disease risk variant in *RPS9/LILRB3*, rs11666543 (*p* = 2.34 × 10^−8^), correlates with reduced mRNA expression of the inhibitory leukocyte immunoglobulin-like receptor gene *LILRB3*. One study proposed that risk alleles in the *IL6* gene and serum level of IL-6 can be used to stratify patients for anti-IL6 treatment (81). Another risk allele, rs6871626, in *IL12B* is shown to influence the expression of *IL-12p70* and *IL-12p40* genes, resulting in enhanced IL-12 signaling that might further contribute to the pathophysiology of TAK (82). Terao et al. showed that interaction of HLA-B*52 and *LILRB1* may be responsible for activation of natural killer (NK) cells by a way of suppression of inhibitory pathways (41).

*FCGR2A/FCGR3A* is a common susceptibility gene locus for TAK and KD. The Fc-gamma receptors (FcγRs) are responsible for interactive relationship between adaptive and innate immune systems. All immune cells possess these receptors. FcγRs can be classified into one high-affinity receptor (FcγRI) and five low-affinity FcγRs (the different isoforms of FcγRII and FcγRIII) (83). Genetic variants of these receptors have been associated with susceptibility to autoimmune, autoinflammatory, and infectious diseases and response to immunotherapy in cancer patients (6, 84–91). Nagelkerke et al. (51) showed that activation of IgG receptors on monocytes and neutrophils plays a role in the pathophysiology of KD. Inhibition of this activation may be a targeted treatment for patients who carry the *FCGR2A/FCGR3A* risk alleles.

### Immunoglobulin A Vasculitis/Henoch-Schönlein Purpura (IgAV/HSP)

IgAV/HSP is a small-vessel vasculitis of the skin, kidney, gastrointestinal tract, and joints. IgAV/HSP has typically a good prognosis because of the self-limiting disease course; however, renal and gastrointestinal involvement can cause morbidity. IgAV/HSP is the most common childhood vasculitis. Diagnosis of IgAV/HSP is based on clinical features; however, skin and kidney biopsy are useful for diagnosis in selected cases.

The first GWAS of IgAV/HSP revealed that the HLA class II region (mainly HLA-DRB1*01 allele) in the European population is associated with the risk for IgAV/HSP, although this finding did not reach genome-wide significance (42). A meta-analysis confirmed that genetic susceptibility to IgAV/HSP is associated with HLA-DRB1*01 and HLA-DRB1*07 variants and suggested that IgAV may be an HLA class II disease (92). Immunoglobulin A vasculitis with nephritis (IgAVN) and IgA nephropathy (IgAN; also known as Berger's disease) are considered related diseases. The chromosome 1q32 locus that contains the complement factor H (CFH) was identified as an IgAN-susceptible locus. Jia et al. showed the contribution of rs6677604 allele in CFH to the regulation of the complement activation in both IgAN and IgAVN (93).

### Giant Cell Arteritis (GCA)

GCA is a systemic vasculitis characterized by granulomatous inflammation of medium- and large-sized vessels and with particular predilection for the aorta and its extracranial branches.

GCA is the most common vasculitis in the elderly (mostly women) population from western countries (94). GCA can cause headaches, visual loss, jaw claudication, painful scalp, impairment of limb arteries, systemic inflammation, and polymyalgia rheumatica. GCA is a potentially devastating disease, with up to 25% of patients developing aneurysmal disease and stroke. First-line treatment of GCA is a high-dose glucocorticoid (GC), and it should be maintained until symptoms are resolved.

GC resistance and adverse effects are main concerns for treatment of patients with GCA (95). Other agents, such as methotrexate and TNF inhibitors, have been used with limited or no evidence of benefit. Recent studies implicated important roles for T_H_1- and T_H_17-driven inflammatory cascades. Results from new therapies with abatacept and ustekinumab have also been promising.

In 2017, the first GWAS in GCA identified the HLA-DRB1*04 as the strongest risk allele (43). This finding implies that GCA is a predominantly HLA class II disease and is distinct from the other large-vessel vasculitis, TAK, which is considered an HLA class I disease. Carmona et al. (43) reported susceptibility alleles in plasminogen (*PLG*) and prolyl 4-hydroxylase subunit alpha 2 (*P4HA2)* genes in 10 different populations of European ancestry at a genome-wide level of significance (rs4252134, *p* = 1.23 × 10^−10^, OR = 1.28; and rs128738, *p* = 4.60 × 10^−9^, OR = 1.32) (43). Both *PLG* and *P4HA2* play a role in the regulation of angiogenesis and vascular remodeling. The plasminogen system is known to regulate several pathways possibly related to the GCA pathogenesis, such as lymphocyte recruitment and production of TNF-α and IL-6 cytokines (96, 97). The *P4HA2* gene is a hypoxia-responsive gene with a role in collagen biosynthesis and composition of ECM. Hypoxia-inducible factor-1 (HIF-1) regulates the expression of *P4HA2* and other genes, such as *VEGF, IL6*, and *MMP9* (98).

## Epigenetics And Epigenome-Wide Association Studies (Ewas) In Primary Systemic Vasculitides

Epigenetic mechanisms including DNA methylation, histone modifications, and non-coding RNAs regulate gene expression, cellular development, differentiation, and activity (99). Epigenomic alterations are not the result of genetic mutations, and they are reversible; however, they can act as integrators of genetic and environmental disease risk factors. Recent work has suggested that epigenome modifications, for example, histone modifications and micro RNA (miRNA) expression, may be used as a biomarker for disease activity and for monitoring disease progression (100). EWAS aim to identify epigenetic variations associated with disease, for example, differentially methylated CpG sites, by performing a hypothesis-free testing across the whole genome (101). Thus far, EWAS have mainly investigated DNA methylation and its association with human diseases. In general, hypermethylation of promoters typically acts to repress gene transcription.

### Kawasaki Disease

*FCGR2A* (IgG receptor gene) encodes the low-affinity immunoglobulin gamma Fc region receptor II-a protein that is expressed on the surface of macrophages, neutrophils, monocytes, and dendritic cells. This protein leads to increased phagocytosis and production of inflammatory mediators. Genetic variants in *FCGR2A* gene have been linked to a genetic risk for KD (100, 102). The first study to examine the DNA methylation array in KD showed that CpG sites within the promoter region of *FCGR2A* were hypomethylated in whole blood cells from KD patients compared with controls, and especially in patients resistant to IVIG treatment (103). Li et al. demonstrated that DNA methylation patterns changed after IVIG treatment in KD, including reversal of the disease-associated hypomethylation in *FCGR2A* (104). Furthermore, interaction of *FCGR2A* and Toll-like receptors (TLR) can induce a proinflammatory response (105, 106). DNA hypomethylation on Toll-like receptors (TLRs) was detected in KD patients, and the methylation level of the TLRs genes was rescued 3 weeks post-IVIG therapy (107).

*FoxP3* acts as both transcriptional activator and repressor in regulatory T cells (Tregs), and *FoxP3*-dependent transcriptional program is epigenetically controlled by histone modifications at its binding site (108). *FoxP3* binds to ~700 genes and a number of miRNAs, including *miRNA-155*, which upregulation likely contributes to proliferative potential of T_R_ cells. *FoxP3* expression in Tregs of patients with KD is affected by *miR-155* and *miR-31*. In patients with acute KD, decrease in FoxP3^+^ Treg might be associated with reduced expression of miR-155 and overexpression of *miR-31*. These effects were partially reversed following the IVIG treatment (109).

Huang et al. (110) showed that the expression of a neutrophil surface molecule, CD177, is higher during acute stage of KD and correlates with epigenetic hypomethylation. They observed that the *CD177* mRNA level diminished sharply after IVIG treatment and were higher in patients with atypical presentation or KD or IVIG-resistant patients.

Chang et al. (111) recently published that gene expression of *PHLPP1* and *MAPK14* is significantly high in KD and is influenced by methylation. They proposed that hypomethylation status and upregulated expressions of *MAPK14* and *PHLPP1* could be candidate biomarkers for the diagnosis of KD.

Hepcidin, encoded by the *HAMP* gene, has a main role in the pathogenesis of inflammation-associated anemia. Hepcidin levels are high in KD patients, and they decrease after IVIG treatment. Huang et al. demonstrated that KD patients had a considerably higher epigenetic hypomethylation of HAMP promoter than controls, which was reversed following therapy with IVIG. They proposed that HAMP promoter hypomethylation and increased hepcidin levels may serve as a biomarker of KD (112). Huang et al. (113) performed the first genome-wide DNA methylation and gene expression assays in blood samples of KD patients. They found that specific CpG markers were hypomethylated in samples from patients with active disease compared to controls and hypermethylated in samples from patients in convalescent phase compared with samples from patients in acute phase. In particular, methylation changes of CpG markers correlated with the expression S100A genes. S100A8 and S100A9 are inflammatory biomarkers that are usually highly expressed in acute and chronic inflammation. Using *in vitro* model, they showed that S100A proteins enhanced leukocyte transendothelial migration of neutrophils. In summary, this study implicates the role of S100A proteins in the pathogenesis of KD (113).

### Behçet's Disease

The first EWAS in BD showed that CD4+ T cells and monocytes extracted from the peripheral blood of BD patients were hypomethylated in genes associated with cytoskeletal remodeling, cell migration, and tissue invasion, such as *RAC1, RGS14*, and *FSCN2*. Some methylation deficiencies return to normal level during disease remission. These findings suggest that epigenetic modifications of cytoskeleton-related genes are important in the pathogenesis of BD (114). Hypermethylation of the IL-4, *TGF-*β, and GATA binding protein 3 (*GATA3*) in CD4+ cells was noted in patients with active BD, but this may not be disease-specific (115, 116). Conversely, treatment with corticosteroids and cyclosporine (CsA) has beneficial effects by decreasing the methylation level of *TGF-*β and *GATA3* (115). A systematic analysis of miRNA expression profiles and pathway analyses in BD identified a specific miRNA signatures in active disease that regulated the *IFN*γ, *TNF*, and *VEGF-VEGFR* signaling cascades (117). Woo et al. (118) observed differential expressions of miRNAs miR-638 and miR-4488 to be associated with elevated production of IL-6. Activation of the Notch pathway in active BD disease and its association with decreased expression of mir-23 and higher Th17 response have been observed (119). Two functional variants, rs2910164 (miR-146a) and rs11614913 (miR-196a2; Ets-1), confer the risk for BD in Han-Chinese by regulating production of proinflammatory cytokines (120, 121).

The differentially expressed genes identified by EWAS might be good candidate biomarkers for monitoring the disease activity and might represent promising candidates for design of novel therapeutic strategies in BD.

### ANCA-Associated Vasculitis

Normally, the expression of *MPO* and *PRTN3* genes (encoding MPO and PR3 autoantigenes) in neutrophils occurs only in the early stages of cell maturation; however, *MPO* and *PRTN3* are found continuously expressed in neutrophils and monocytes of patients with AAV (122). This deregulation in gene expression is postulated to be related to epigenetic mechanisms (123–125). Jones et al. (124) showed that the gene-specific DNA methylation changes may regulate autoantigen expression, and they correlate with disease activity in AAV. In remission, DNA methylation is generally increased. Patients with active disease had hypomethylation and increased expression of *MPO* and *PRTN3*. The findings suggest that the reactivation of once-silenced genes can lead to increased antigen availability. Patients with decreased DNA methylation at the *PRTN3* promoter have a significantly greater risk of relapse. Therefore, fluctuations in the DNA methylation of the *PRTN3* promoter may be associated with stable remission. Methylation status may prove to be a potential biomarker for prognosis in AVV patients (124).

### Immunoglobulin A Vasculitis/Henoch-Schönlein Purpura

Luo et al. (126) observed that global histone H3 acetylation and H3K4 methylation are increased in peripheral blood mononuclear cells (PBMCs) isolated from IgAV patients. These epigenetic modifications had positive correlations with disease activity and were more common in IgAV patients with renal involvement compared with IgAV patients without renal involvement and healthy controls. They observed increased mRNA expression of *GATA3, IL-4, IL-6*, and *IL-13* genes in IgAV patients. Serum protein levels of IL-4, IL-6, and IL-13 were significantly increased in HSP with kidney damage patients compared to healthy controls. Abnormal histone modifications of transcription factors such as GATA3 may lead to the Th1/Th2 cytokine imbalance in HSP and other immune diseases (126).

### Giant Cell Arteritis

Only two epigenetic studies evaluated DNA methylation status and miRNA expression in temporal artery biopsies (TABs) of patients with GCA (127, 128). Coit et al. (127) identified 1,555 hypomethylated CpG sites in 853 genes by comparing 12 patients and 12 healthy age-, gender-, and ancrestry-matched controls. DNA was extracted from the affected temporal artery tissues in patients with GCA and from histologically confirmed normal arteries in controls.

Most of these genes have roles in T-cell activation and differentiation pathways, especially Th1 and Th17 cells. *TNF, LTA, LTB, CCR7, RUNX3, CD6, CD40LG, IL2, IL6, NLRP1, IL1B, IL18, IL21, IL23R*, and *IFNG* were proinflammatory hypomethylated genes that were found in this study. Interestingly, the significant hypomethylation of genes in the calcineurin (CaN)/NFAT pathway was shown within the temporal artery of GCA patients.

Increased activity in NFAT is one of the key factors in production of proinflammatory cytokines. This study provided evidence for use of NFAT inhibitors (e.g., dipyridamole) in the treatment of GCA (127, 129).

Croci et al. showed that miR-146b-5p,−146a,−21,−150,−155, and−299-5p are expressed at higher levels in affected vessels of TAB-positive GCA patients compared to non-inflamed TABs from GCA patients and to non-inflamed TABs from non-GCA patients. Interestingly, these miRNAs were mainly deregulated at the tissue level, whereas no difference in their expression was observed in peripheral blood mononuclear and polymorphonuclear cells from these three groups of patients and controls. Especially, miR-146b-5p expression was the most promising diagnostic biomarker for the presence of inflammation in TABs of GCA patients and the follow up of the disease activity. Further research is necessary to optimize their use in medical practice (128).

## Extracellular Vesicles In Primary Systemic Vasculitides

EVs were defined in 1967 as “dust” from platelets (130). More recently, it has been shown that EVs are membrane vesicles that are released by almost all eukaryotic cells during cell activation and programmed cell death (131–134). Heterogeneity of EVs is essential. They are classified into three groups with regard to biological features and their size: exosomes, microvesicles (MVs), and apoptotic bodies (132, 133, 135, 136). EVs are responsible for immune regulation, cell-to-cell interaction, and signal transmission by transporting bioactive molecules including proteins and lipids, DNA, and various RNAs, such as mRNAs, small-interfering RNAs (siRNAs), microRNAs (miRNAs), and long non-coding RNAs (lncRNAs) and other molecules produced by cells (132, 137–139). EVs can be detected in many organs, tissues, and all body fluids, such as urine, blood, and synovial fluid at low levels in physiological conditions (140, 141). The increased levels of EVs are noted in cardiovascular disease, cancer, and pathological conditions that are associated with vasculitis: inflammation, autoimmunity, endothelial damage, angiogenesis, procoagulation, and intimal hyperplasia (131–133, 136, 140–142). EVs are known to be released from injured endothelial cells and are found increased in many diseases associated with endothelial dysfunction. It is thought that EVs from platelets, lymphocytes, and monocyte/macrophages contribute to the pathogenesis of systemic vasculitis (143–170) (Table 3).

**Table 3:** The role of extracellular vesicles in vasculitis. This table is adapted from Wu et al. (133).

**Author and references**	**Origin of EVs**	**Content of EVs**	**Related diseases and vessels**	**Possible pathogenic mechanism**
**Microvesicles**				
Nakaoka et al. (147)	Endothelial cells, CD144	miR-145-5p, miR-320a	KD, medium vessels	Upregulation of proinflammatory cytokine
Raposo et al. (136)	Leukocytes CD45	B1-receptors	AAV, IgAV, small vessels	Kinin system
Raposo et al. (136)	Endothelial cells	B1-receptors	AAV, IgAV	Kinin system
Macey et al. (153)	Platelets CD42a/CD62P	–	BS	Inflammation
Dursun et al. (144)	Endothelial cells	–	IgAV/small vessels	Inflammation
Daniel et al. (167)	Neutrophils CD66b	–	AAV	Inflammation and procoagulation
Mendoza-Pinto et al. (171)	Neutrophils annexin	Tissue factor	AAV	Inflammation and thrombosis
Hong et al. (172)	Annexin V neutrophils	CD18/C, D11b, PR3, MPO	AAV	Inflammation and procoagulation
Huang et al. (161)	Annexin V	Tissue factor	AAV	Procoagulation
Mejia et al. (155)	Annexin V	–	BD	Procoagulation
Khan et al. (152)	Annexin V	Tissue factor	BD	Procoagulation
Eleftheriou et al. (145)	Annexin V, platelets CD41 endothelial cells CD62E	Tissue factor	MPA, GPA, PAN, KD, BS	Procoagulation
Martinez et al. (154)	Platelets CD6	–	BS	Procoagulation
Yahata et al. (170)	Platelets CD42b/CD42a	–	KD	Evaluation of platelets
Kim et al. (169)	Annexin V	–	KD	Evaluation of platelets
Hajj-Ali et al. (146)	Annexin endothelial cells CD105/CD144, platelets CD41, leukocytes CD18, neutrophils	–	GPA/small vessels	Platelets activation and endothelial damage
Tian et al. (166)	Annexin V V/CD62E/CD31	–	KD/medium vessels	Endothelial damage
Erdbruegger et al. (168)	Annexin V, endothelial cell CD105/CD6 2E	–	AAV/small vessels	Endothelial damage
Ding et al. (163)		–	KD/medium vessels	Endothelial dysfunction
Kumpers et al. (165)	Annexin V	–	CSS/small vessels	Endothelial damage
Clarke et al. (162)	Annexin V, endothelial cell	–	MPA, GPA, PAN, KD, BS	Endothelial damage
Guiducci et al. (160)	Platelets CD42, erythrocytes, T cells, endothelial cells	–	KD/medium vessels	Endothelial damage
Brogan et al. (143)	Annexin V, platelets CD42a/CD62	–	MPA, GPA, PAN, KD, BS	Endothelial activation
Tan et al. (149)	Endothelial cells CD31/CD146	–	KD/medium vessels	Endothelial damage
Shah et al. (148)	Endothelial cells CD105/CD62E	–	KD/medium vessels	Endothelial damage
**Exosomes**				
Jia et al. (150)	CD9/CD81/TS	miR-1246, miR44	KD/medium vessels	Diagnostic biomarker
Zhang et al. (159)	–	miR-328, miR-575, miR- 134, miR-671- 5p	KD/medium vessels	Inflammation
Zhang et al. (158)	CD9/CD81/flotilin	38 different contents	KD/ medium vessels	Inflammation and procoagulation
Zhang et al. (157)	CD9/flotillin	69 different proteins	KD/medium vessels	Inflammation and procoagulation

In 2004, Brogan et al. (143) reported that the endothelial microparticles expressing E-selectin-positive or CD105 and the platelet MVs expressing CD42a were significantly increased in patients with active vasculitis compared to controls or children with inactive vasculitis or other febrile illnesses. They found that endothelial microparticle levels correlate with the Birmingham Vasculitis Activity Score (BVAS) and acute-phase reactant levels (143).

Activation of the contact system has proinflammatory and vasoactive properties. The contact system triggers the kallikrein-kinin cascade releasing bradykinin from high-molecular-weight kininogen. Bradykinin concentrations were higher in the patients' plasma than in plasma from controls, and kinins were present in lesional biopsy tissues of kidney and skin of children with PSVs (173, 174). The kinin B1 receptor is induced by inflammatory stimuli, particularly in response to the cytokine IL-1β, and plays a major role in neutrophil recruitment. Kahn et al. (151) found that leukocyte-derived MVs transfer functional kinin B1-receptors and in this way may further promote kinin-mediated inflammation in vasculitis. Mossberg et al. (156) showed that patients with acute vasculitis had significantly higher levels of circulating endothelial MVs and more MVs carrying B1-receptors. These MVs can induce the inflammation by increasing neutrophil chemotaxis, whereas addition of a C1-inhibitor decreased the release of B1R+MVs. Kinin-B1 receptor activation of CXCL5 can further amplify neutrophil chemotaxis and chronic inflammation (175). Exploring inhibition of the kallikrein-kinin pathway is potentially a novel target for reducing neutrophil-mediated inflammation in PSVs.

### Kawasaki Disease

Endothelial cell-derived MVs were detected in higher levels in children with KD (147–149, 163, 166) and HSP (144). These MVs may serve as biomarkers to predict coronary artery aneurysms in KD and to detect subclinical inflammation in HSP (144, 148).

The role of platelet activation dynamics in acute-phase KD patients was explored by assaying platelet-derived MVs (PDMVs). Prior to aspirin treatment, PDMV level was significantly higher in the acute-phase KD patients in comparison to patients with common febrile diseases. Guiducci et al. observed that MVs derived from platelets, endothelial cells, erythrocytes, and T cells are significantly elevated in plasma samples of patients with KD. Endothelial and T cells were the major source of MVs, and the levels were reduced by IVIG treatment (160). Platelet activation is important in the pathogenesis of KD, thus PDMVs may serve as a biomarker to evaluate the antiplatelet therapy response in KD (169, 170).

Measuring microRNAs (miR-4436b-5p, miR-1246, miR-671-5p, and miR-197-3p) in serum exosomes has been proposed as a diagnostic biomarker for the prediction of IVIG response in KD (149, 159). Serum exosomal microRNAs may have a role in the pathogenesis of KD by regulating the expression of inflammatory genes (CX chemokine receptor types 1 and 2, IL-8, and its receptor) in mononuclear cells (159). Serum exosomes from patients with KD contain many proteins, such as apolipoprotein A-IV, insulin-like growth factor-binding protein complex, acid-labile subunit and complement C3, and their proteomic profile correlates with IVIG therapy (157). Zhang et al. performed proteomic analyses of serum exosomes from KD patients with coronary artery dilatation. They found 38 differentially expressed proteins, and the majority are involved in inflammatory and coagulation pathways (158). More recent proteomic analysis validated TN, APOA4, LRG1, and RBP4 proteins as differentially expressed in patients with coronary artery aneurysms (CAA) (176). These finding provide additional insights in the pathogenesis of CAA in patients with KD.

### ANCA-Associated Vasculitis

Endothelium-derived MVs have been reported in active AAV patients (168). Plasma levels of MVs derived from platelet and neutrophil are high in acute-phase vasculitis (167). The neutrophil-derived MVs cause increased production of proinflammatory cytokines IL-6 and IL-8, increased expression of intercellular adhesion molecule-1, and production of reactive oxygen radicals by binding to endothelial cells in a CD18-dependent manner (172). Furthermore, endothelial MVs carry PR3 and MPO and may contribute to the extensive endothelial damage and inflammation seen in AAV (177, 178).

Thromboembolic disease complicating primary systemic vasculitis is associated with significant morbidity and mortality. The mechanisms of hypercoagulability in PSV remain poorly defined. Several studies attempted to identify risk factors of thrombosis to stratify patients who could benefit from prophylaxis with antiplatelet or anticoagulant agents. One study showed that stimulation with ANCAs causes C5a-primed neutrophils to release neutrophil tissue factor (TF)-expressing MVs and neutrophil extracellular traps (NETs) that might promote hypercoagulability in AAV (161). Eleftheriou at al. have demonstrated that thromboembolic disease in children with systemic vasculitis is linked to increased level of MVs-mediated thrombin (145).

### Behçet's Disease

Thrombosis is common in BD patients, and there is a need for better assessment of risk factors. BD patients with a history of thrombosis have high serum levels of MVs expressing tissue factor (TF). The ratio of TF pathway inhibitor (TFPI)-positive MVs to TF-positive MVs was significantly lower in patients with thrombosis (152). In contrast, platelet-derived MVs and procoagulant MVs did not differentiate between BD patients with or without thrombosis (155). Macey et al. (153) showed that high-level platelet-derived CD62P+ MVs correlate with active disease in patients in a younger age group, whereas lower levels of MVs correlate with decreased disease activity in patients older than 50 years.

## Insights From Studies of Monogenic Systemic Autoinflammatory Diseases

Rare Mendelian diseases of systemic inflammation often present with severe-like phenotypes of polygenic diseases and can share underlying biochemical pathways with more common rheumatic diseases. Identification of patients with monogenic disease can point to genes and pathways that could be investigated in patients with polygenic disorders. For instance, polyarteritis nodosa (PAN) can present either as early-onset monogenic or late-onset polygenic disease. PAN is a systemic necrotizing vasculitis that predominantly affects medium-sized arteries, causing tissue ischemia and organ damage. The disease commonly affects the skin, gastrointestinal tract, and kidneys, and patients are at an increased risk of stroke. Most cases of PAN occur in the fourth or fifth decade, and men are more likely to be affected. A subset of patients with childhood-onset PAN are found to carry biallelic loss of function mutations in adenosine deaminase 2 gene. This disease is named deficiency of ADA2 (DADA2)(179, 180). Among many features of DADA2, subcortical ischemic strokes, hypertension, aneurysms, renal infarcts, and peripheral amputations have been reported in up to 50% of patients. DADA2 patients are highly responsive to treatment with TNF inhibitors (181). Although pathogenic variants in the *ADA2* gene may not account for a large number of sporadic adult-onset PAN patients, TNF inhibitors should be explored in the treatment of those patients as well (181).

### Inflammasomopathies

Inflammasomopathies are rare monogenic autoinflammatory diseases caused by gain-of -function mutations in the multiprotein complexes termed the inflammasome. The inflammasome functions as cytosolic pathogen and danger-associated molecular patterns (PAMPs/DAMPs) recognition receptors (PRRs). The core of inflammasome is one of the nucleotide-binding domain leucine-rich repeat (NLR) proteins (NLRP1, NLRP3, AIM2, NLRC4) or pyrin. Upon stimulation, the inflammasome interacts with an inflammasome adaptor protein, apoptosis-associated speck-like protein with a caspase recruitment domain (ASC), or with pro-caspase 1 to form platform for caspase-1-mediated production of IL-1β and IL-18 cytokines. The best known inflammasomopathies are familial Mediterranean fever (FMF) and cryopyrinopathities (CAPS). FMF is caused by recessively inherited hypomorphic mutations in pyrin, whereas CAPS is linked to dominantly inherited gain-of-function mutations in NLRP3. The net effect of these pathogenic variants is increased production of IL-1β cytokine and systemic inflammation. FMF is a common disease in many Eastern Mediterranean countries where the frequency of pathogenic mutations is very high (182–205). Although vasculitis is not a primary feature of FMF, IgAV and PAN have been reported in about 3 and 1% of FMF patients, respectively (206). FMF-associated mutations predispose to the development of BD in the Turkish population (207–210). Small and medium vessel skin vasculitis and CNS vasculitis were reported in other inflammasomopathies, including CAPS and other pyrin-mediated autoinflammatory diseases mevalonate kinase deficiency/hyperimmunoglobulin D (MKD/HIDS); pyogenic arthritis, pyoderma gangrenosum, and acne (PAPA); and pyoderma gangrenosum, acne, hidradenitis suppurativa (PASH) syndromes (211–221). NLRP3 inflammasome activation was demonstrated in two Kawasaki mouse models (222, 223). Underlying mechanisms of vasculitis in inflammasomopathies are unclear, although it is assumed that high systemic IL-1β production may cause endothelial cell inflammation and damage. Patients with BD may respond to colchicine and anti-IL1 therapies like patients with inflammasomopathies (224).

### Relopathies

Relopathies are NF kappa-B (NF-κB)-mediated monogenic systemic autoinflammatory diseases driven by multiple cytokines including TNF and IL-6 (225). They result from pathogenic variants in proteins that regulate posttranslational ubiquitin modifications in the NF-κB pathway. Up to now, haploinsufficency of A20 (HA20) and OTULIN deficiency have been defined as relopathies (226). HA20 is a dominantly inherited disease caused by heterozygous loss-of-function mutations in *TNFAIP3*, which encodes the K-63 deubiquitinase protein A20 (227). Common variants in *TNFAIP3* have been linked to BD in the Chinese-Han population (228). Clinically, HA20 resembles early-onset BD. Two patients with HA20 were diagnosed with CNS vasculitis based on brain imaging and a frontal lobe punctate (229). OTULIN deficiency is caused by recessively inherited loss-of-function mutations in the enzyme that regulates linear deubiquitination. There are only few patients reported, and they present with early-onset severe systemic inflammation. One patient was described with vasculitis of small and medium vessels on skin biopsy (230). In most patients with HA20 and OTULIN deficiency, anti-cytokine therapies targeting TNF and IL-1 are efficient in suppressing the disease activity. Ubiquitin pathway has been implicated in the pathogenesis of BD by multiple association studies. The rs9517723 variant in the 3' region of ubiquitin-associated domain containing ubiquitin-associated domain containing 2 *(UBAC2)* gene is significantly associated with ocular and central nervous system (CNS) lesions under the recessive model (32, 231). *UBAC2* encodes a protein that plays a role in ubiquitination and proteasomal degradation. Homozygous risk allele (TT) of the rs9517723 correlates with increased *UBAC2* expression. BD has been also associated with other ubiquitination pathway-related genes, including ubiquitin associated and SH3 domain containing B (*UBASH3B*) and small ubiquitin-like modifier 4 (*SUMO4*) (232, 233).

### Interferonopathies

Type I interferonopathies consist of Aicardi–Goutières syndrome (AGS), STING-associated vasculopathy with onset in infancy (SAVI), chronic atypical neutrophilic dermatosis with lipodystrophy and elevated temperature (CANDLE) syndrome, and COPA syndrome (226). Intracerebral vasculitis is common in patients with AGS (234). High levels of IFN activities were demonstrated in the cerebrospinal fluid and serum of AGS patients (234). In SAVI, gain-of-function mutations in the *TMEM173* gene lead to a constitutively active STING protein, and a high expression of type I IFN-induced genes (235, 236). Clinically, the SAVI phenotype mimics the AAV with cutaneous rashes, interstitial lung disease, peripheral ulcerations/gangrene, and ANCA positivity in some patients (237). Patients with CANDLE have dysfunction in the protesome-mediated degradation pathway. Skin biopsies from CANDLE patients showed evidence for perivascular infiltration prominent for myeloid cells and leukocytoclasis but not for the typical vasculitis with fibrinoid necrosis of the vessel walls (238). JAK1/2 inhibition with baricitinib has been successful in treating clinical disease manifestations and in suppression of the IFN signature (239). The role of the IFN pathway in the pathogenesis of polygenic/complex vasculitides has been recently postulated by analysis of data from multiple GWAS (78).

Reports of vasculitis in other rare autoinflammatory diseases are scarce. One patient with the deficiency of IL-1 receptor antagonist (DIRA) had histopathological evidence of vasculitis in the connective tissue and cerebral vasculitis or vasculopathy was detected on magnetic resonance imaging (240). IL-1Ra-deficient mice frequently develop aortitis at the root of the aorta, which can be suppressed by a cross with TNF-α-deficient mice. Bone marrow transplantation of T cells from IL-1Ra^−/−^ mice induced aortitis in recipient nu/nu mice or in irradiated wild-type recipient mice (241). This study demonstrates that IL-1Ra deficiency in T cells is responsible for the development of aortitis.

## Expert Commentary

- The main limitations of current genetic datasets in PSVs are the lack of multiple meta-analyses of GWAS and inability to confirm functional significance of identified risk alleles. Validation of disease-associated variants in cohorts of patients of different ancestry would give more creditability to the reported findings; however, some genetic variants may not be present in all populations.
- As epigenetic modifications evolve and change over the life span, the data from EWAS will require validation in longitudinal cohort studies that are costly to maintain. Epigenomic investigations maybe accomplished in a shorter time period if they focus on studies in monozygotic twin pairs (MZ pairs) or if they would investigate differences in young vs. old MZ pairs. In addition, it would be interesting to explore cell-specific epigenomic modifications.
- Use of extracellular vesicles (MVs, PDMVs, EMVs) as potential biomarkers of disease activity in patients with primary systemic vasculitis is important for diagnostic and treatment monitoring. At present time, there are no standardized methods for the sampling process and detection of EVs.

## 5-Year Forward View

Multicenter collaborations and studies in large cohorts of patients would increase the power of detection and confirmation of PSV disease-associated variants. These meta studies would also help estimate a size effect of identified risk alleles, which is important for calculation of genetic risk score (GRS) and genetic counseling. Considering a significant decrease in the cost of whole exome and whole genome sequencing (WES/WGS), these new sequencing technologies should capture rare germ-line variants, structural variants, and mosaic mutations that may explain the missing heritability in these diseases. At this time, it is still unclear whether SNPs captured by GWAS are true disease-associated variants or in linkage disequilibrium with a risk allele. Performing WGS of patient cohorts would avoid a need for imputation studies. Effects of most common disease-associated variants on protein function are unclear, in particular for non-coding variants. Disease-associated common variants may have very small effect sizes but could lead to important and targetable pathways. Studies in cell culture systems and model organisms and experiments in primary patient cells are needed to answer this question.

## Conclusion

Management of PSVs and other human diseases is beginning to be more directed with increased use of molecular data to improve diagnosis and to guide optimal treatment options. This new clinical paradigm has premise to apply knowledge of genomic, proteomic, and metabolic variants and epigenomic biomarkers to generate “omic” profiles. The translation into clinical practice can be achieved by integrating the “omic” information into a unique algorithm that will be able to make accurate diagnosis and to optimize therapeutic decisions to maximize benefit and minimize harm. Proper implementation of PM will require multidisciplinary teams of clinicians, bioinformaticians, geneticists, genetic counselors, and research scientists that can address multiple challenges in interpretation and integration of the multi-omics results. Along these lines, successful transition toward personalized care will also necessitate updating medical curriculums to facilitate training of a new generation of “omic”-literate physicians.

## Author Contributions

ED and ZA drafted the manuscript. MR and RB revised the manuscript and designed the tables. IA revised and finalized the manuscript.

### Conflict of Interest

The authors declare that the research was conducted in the absence of any commercial or financial relationships that could be construed as a potential conflict of interest.
